# Signatures of historical selection on MHC reveal different selection patterns in the moor frog (*Rana arvalis*)

**DOI:** 10.1007/s00251-017-1051-1

**Published:** 2018-02-01

**Authors:** M. Cortázar-Chinarro, Y. Meyer-Lucht, A. Laurila, J. Höglund

**Affiliations:** Animal Ecology/Department of Ecology and Genetics, Uppsala University, Norbyvägen 18D, 75236 Uppsala, Sweden

**Keywords:** Directional selection, Major histocompatibility complex, *Rana arvalis*, Codon models

## Abstract

MHC genes are key components in disease resistance and an excellent system for studying selection acting on genetic variation in natural populations. Current patterns of variation in MHC genes are likely to be influenced by past and ongoing selection as well as demographic fluctuations in population size such as those imposed by post-glacial recolonization processes. Here, we investigated signatures of historical selection and demography on an MHC class II gene in 12 moor frog populations along a 1700-km latitudinal gradient. Sequences were obtained from 207 individuals and consecutively assigned into two different clusters (northern and southern clusters, respectively) in concordance with a previously described dual post-glacial colonization route. Selection analyses comparing the relative rates of non-synonymous to synonymous substitutions (dN/dS) suggested evidence of different selection patterns in the northern and the southern clusters, with divergent selection prevailing in the south but uniform positive selection predominating in the north. Also, models of codon evolution revealed considerable differences in the strength of selection: The southern cluster appeared to be under strong selection while the northern cluster showed moderate signs of selection. Our results indicate that the MHC alleles in the north diverged from southern MHC alleles as a result of differential selection patterns.

**Electronic supplementary material:**

The online version of this article (10.1007/s00251-017-1051-1) contains supplementary material, which is available to authorized users.

The major histocompatibility complex class II plays a pivotal role in binding and presenting peptides derived from extracellular pathogens for recognition on CD4+ lymphocytes and T cell receptors (Janeway et al. 2017). The antigen presentation will elicit an appropriate immune response in the host based on the type of MHC class II molecule coded by different class II alleles (Wood 2006). The MHC class II exon 2 is by far the most polymorphic and diverse coding genetic region in most vertebrates (Hughes 1999, Klein 1986), and variation is especially pronounced in the peptide binding regions (PBRs; Bernatchez and Landry 2003; Piertney and Oliver 2006). This high MHC variability is beneficial for the host in the face of newly emerging diseases (Schemske et al. 2009). As a survival strategy, pathogens evolve to escape being recognized by the most frequent host MHC molecules, which might lead to infections in large parts of the population and ultimately increase the risk of extinction (Hedrick 2002; Wood 2006). As a consequence of this arms race, host populations may express hundreds of different MHC variants, making it more difficult for pathogens to fully avoid detection (Kohn et al. 2006; Wood 2006).

The molecular structure of the MHC has been extensively studied in humans and many other mammalian species (Janeway 2001). However, the MHC structure in other vertebrates, such as amphibians, is less well known. To study the MHC structure in other taxa, researchers commonly compare the amino acid positions of PBRs of the human leukocyte antigen (HLA; the human MHC; Bondinas et al. 2007; Brown et al. 1993; Tong et al. 2004) to infer sites involved in peptide binding. The variability at the PBR is believed to be subject to strong selective pressure and contains signatures of both positive and negative selection (Meyer and Thomson 2001).

In Scandinavian moor frogs (*Rana arvalis*), genetic variation at the MHC class II exon 2 is shaped by a complex pattern of past and ongoing selection, drift, and/or historical demographic events including post-glacial recolonization history where selection is a relatively more important process in the south whereas drift is more important in the north (Cortázar-Chinarro et al. 2017). These processes can increase or decrease genetic variation and explain the observed patterns of current genetic variation in nature (Hedrick 1998). Several studies have found that MHC II exon 2 can be under divergent selection among different populations, suggesting adaptation to differences in the local parasite fauna (e.g., Ekblom et al. 2007; Meyer-Lucht et al. 2016). Others have found patterns indicating long-term balancing selection maintaining trans-species polymorphism (e.g., Vlček et al. 2016). Furthermore, other authors showed positive selection and trans-species polymorphism working at the same time (Surridge et al. 2008). However, none of the studies have explicitly examined the relationships between the patterns of historical selection at MHC at latitudinal scales, and how MHC variation can be shaped by drift, selection, and colonization processes.

Amphibian populations are suffering worldwide declines and are the most threatened vertebrate taxon on the planet (Stuart et al. 2004). Habitat fragmentation in combination with infectious diseases, such as chytridiomycosis caused by the fungus *Batrachochytrium dendrobatidis* (Bd), has been identified as the most important factor in the amphibian decline (Berger et al. 1998; Garner et al. 2006; Lips et al. 2006; Morgan et al. 2007). A number of studies in amphibians suggest that specific MHC class II molecules are involved in immunity against Bd and other disease agents (Babik et al. 2008, 2009; Lillie et al. 2015; Zeisset and Beebee 2009, 2013), suggesting a link between MHC II variability and the susceptibility or resistance to fungi and other disease agents (Bataille et al. 2015; Savage and Zamudio 2011).

In this study, we explore the mode of historical selection in 12 *R. arvalis* populations along a 1700-km latitudinal gradient from northern Germany to northern Sweden. Previous phylogeographic studies using microsatellites, mtDNA, and allelic variation at the MHC II exon 2 gene in *R. arvalis* indicate two post-glacial recolonization routes into Scandinavia (Babik et al. 2004; Cortázar-Chinarro et al. 2017; Knopp and Merilä 2009). Under this scenario, populations were assigned belonging to either of two different clusters (northern and southern clusters, respectively) suggesting that individuals from the two lineages evolved in allopatry until they came into secondary contact ca. 5000 years ago. Our ultimate goal is to investigate the mechanisms underlying the historical formation and maintenance of MHC II exon 2 variation along the two post-glacial colonization routes.

We used the MHC class II exon 2 data set described in Cortázar-Chinarro et al. (2017) for analyzing signatures of historical selection in natural populations of the moor frog (*R. arvalis*) along the latitudinal gradient (see supplementary material; Table S1). DNA extraction methods, primer design, MHC genotyping, and data filtration are detailed in Cortázar-Chinarro et al. (2017). In this previous study, we assessed contemporary selection primarily based on general allelic frequencies and a classical F_ST_ outlier approach. We identified the MHC II exon 2 (corresponding to the β-2 domain) to be subject to diversifying selection, while five microsatellite loci showed signals of stabilizing selection among populations. In general, contemporary signs of selection acting on MHC were weaker in the north, possibly as a consequence of the effects of demography. In the present study, we conduct calculations for inferring signatures of historical selection on the MHC sequences, dN/dS, ratios and Tajima’s D, as well as dN/dS per codon in a sliding window approach on each position, looking at selection from a molecular point of view.

We used MEGA v.7.0 to visualize the data and align haplotype sequences of the MHC class II gene exon 2β chain (Kumar et al. 2016). To determine whether the sequences constitute classical functional class II alleles, we looked for the presence of indels causing a shift in the reading frame and/or stop codons. The peptide binding region was defined according to the human HLA (Bondinas et al. 2007). Some of the codons are generally highly conserved among species in classical MHC class II molecules (Bondinas et al. 2007; Brown et al. 1993). We used the MHC class II exon 2 (272 bp) data set from Cortázar-Chinarro et al. (2017) for analyzing natural populations of the moor frog (*R. arvalis*; total 207 individuals, 12 populations) along a latitudinal gradient (77 individuals from the north, Umeå and Luleå, and 130 from the south, Germany, Skåne, and Uppsala, Table S1). We amplified and sequenced the complete exon 2 (272 bp) at a single MHC II locus. We detected 57 unique haplotypes over the entire gradient; 4 haplotypes were shared between the northern and southern cluster (Table 1; Fig. 1; see Cortázar-Chinarro et al. 2017). Each of the 57 haplotypes translated into 90 amino acids, none of the allele sequences contained an indel or a stop codon, and we thus assume that they all are functional.

**Table 1:** Genetic diversity measures. Number of individuals (*N*), number of alleles (*A*), number of private alleles (Ap), number of segregating sites (*S*), allelic richness (AR), Observed heterozygosity (*H*_o_), expected heterozygosity (*H*_e_), inbreeding coefficient (*F*_IS_), average number of pairwise differences (theta K), nucleotide diversity (П), neutrality test summary (Tajima’s *D*)

	*N*	A	Ap	S	AR	*H*_o_	*H*_e_	*F*_IS_	*k*	П	Tajima’s D
Entire gradient	207	57		31	38	0.561*	0.762	0.264	10.731	0.039	1.007
Northern cluster	77	14	6	19	6	0.470	0.544	0.136	4.407	0.016	− 1.251
Southern cluster	130	47	30	31	12	0.613*	0.806	0.239	11.739	0.043	1.289

*Significant deviation from Hardy-Weinberg (*p* < 0.001)

**Fig. 1:**
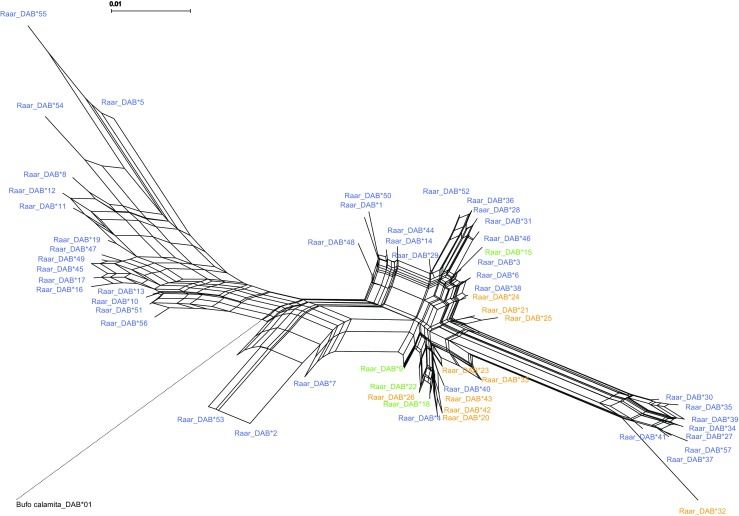
Neighbor network for the 57 MHC II exon 2 sequences in *R. arvalis*. Blue represents sequences from the southern cluster, orange from the northern cluster, and green represents sequences shared between northern and southern clusters. A natterjack toad sequence [Genbank HQ388291.1] from MHC II exon 2 was used as the outgroup

Nucleotide diversity (П), number of segregating sites (*S*), and average number of pairwise nucleotide differences (theta K) were calculated in DNAsp v5.0 (Librado and Rozas 2009). Observed heterozygosity (*H*_O_), expected heterozygosity (*H*_E_), and allelic richness (AR) were calculated in Fstat (Goudet 2001). Deviations from Hardy-Weinberg equilibrium were tested for in Genepop (Rousset 2008). All the genetic diversity measures were calculated for the entire gradient, and for the northern and the southern cluster separately. The number of segregating sites (*S*) was 31 (11% of the 272 nucleotide sites) in the entire gradient and in the southern cluster, and 19 in the northern cluster (7% of the 272 nucleotide sites). MHC II exon 2 revealed a higher nucleotide diversity (П) and higher average number of pairwise differences (theta K) in the entire gradient and in the south compared to the north (Table 1).

We constructed a phylogenetic tree to illustrate the phylogenetic relationship among *R. arvalis* MHC sequences using the Neighbor joining method with bootstrapping (1000 replicates, Fig. S1) implemented in MEGA v7.0 (Kumar et al. 2016). We also reconstructed an unrooted phylogenetic network in the software SplitTree4, using the neighbor net method (Huson and Bryant 2005, Fig. 1). In both analyses, we included a natterjack toad (*Epidalea calamita*; GenBank: HQ388291.1) sequence as an outgroup. We further constructed a haplotype network in the *Hapstar* software (Teacher and Griffiths 2011), which shows the number of base-pair changes over the sequences (Fig. S2). The input file was generated by selecting the minimum spanning tree option in ARLEQUIN software (Excoffier and Lischer 2010). Statistical network analyses were run with the “igraph” package in R (Csardi and Nepusz 2006). All three approaches showed that the MHC II exon 2 sequences from the northern groups are clearly non-randomly distributed within the network, indicating their sequence similarity among each other (Figs. 1, S1, and S2). Only four out of the 57 haplotypes were shared between the northern and southern clusters (Raar_DAB*15, Raar_DAB*18, Raar_DAB*20, Raar_DAB*22). Among these four haplotypes, Raar_DAB*15 was found in nine out of 12 populations along the gradient and it is central in the haplotype network (Fig. S2).

We used a number of approaches to investigate whether regions of the *R. arvalis* MHC class II sequences carry signatures of selection (i.e., over evolutionary time), either as positive or negative selection (Piertney and Oliver 2006). Positive selection is the process by which new advantageous alleles are spread to fixation and therefore increase the fitness of individuals in a population. In contrast, negative selection is the selective removal of deleterious alleles which ultimately reduces variation in a population. In MHC genes, positive selection may suggest adaptive responses to pathogen diversity while negative selection or purifying selection may also suggest that the MHC fragment plays a functional role independent of diseases or microbial resistance by removal of deleterious alleles in a population and reducing the frequency of mutations in a pool of linked genes (see e.g., Charlesworth 2006).

We used DNAsp (Librado and Rozas 2009) to calculate Tajima’s D, a statistical method for testing for deviation from neutral evolution of DNA sequences, on the MHC II exon 2 locus. While we detected negative Tajima’s D values in the northern cluster, and positive values of Tajima’s D in the entire gradient and southern clusters (Table 1), the lack of significance (*p* > 0.10) indicates that deviation from neutrality could not be detected by this approach.

To detect historical selection (i.e., over evolutionary time), we used a common method based on the ratio of non-synonymous to synonymous nucleotide substitutions (dN/dS or *ω*). Ratios of dN/dS > 1 indicate positive selection and dN/dS < 1 indicate negative purifying selection (Garrigan and Hedrick 2003; Hughes 1999, 2007). We used two different methods to calculate ratios of synonymous to non-synonymous substitutions. In the first approach, we estimated the average synonymous (dS) and non-synonymous (dN) substitutions per synonymous and non-synonymous site as implemented in MEGA v.7.0 (Kumar et al. 2016). We used the Nei-Gojobori/Jukes-Cantor method with 5000 bootstrap replicates to calculate the overall average of dN/dS (*ω*) for all sites, the peptide binding region (PBR), and non-PBR according to Bondinas et al. (2007). Deviations from neutrality (dN ≠ dS) were assessed by a *Z* test. Differences in dN and dS between PBR and non-PBR were tested for significance using Mann-Whitney *U* tests. In addition, we identified signatures of positive selection using OmegaMap as implemented by Wilson and McVean (2006).

We estimated dN and dS at the PBR and the non-PBR (Bondinas et al. 2007) for the entire gradient, the northern and the southern clusters (Table 2; Fig. S3). For the PBR, the values for the ratio (*ω*) were larger than 1 in all the groups (entire gradient, northern and southern subgroups). The *Z* test for selection indicated that *ω* was significantly different from neutral expectations in all groups (entire gradient Z = 3.894; northern cluster; Z = 2.672 and southern cluster = 3.621; *p* < 0.001) (Table 2; Fig. 2). This is considered as evidence of positive selection acting on the PBR. Codons outside the putative PBR regions showed a *ω* value < 1 in the entire gradient and in the south, suggesting negative selection; however, neither of these were significant (Table 2). In the north, *ω* was > 1, but not significant (Table 2). Values of *ω* > 1 may indicate positive selection and/or effects of demography.

**Table 2:** Relative rates of non-synonymous (dN) and synonymous (dS) substitutions, the peptide binding region (PBR), and non-PBR according to Bondinas et al. (2007). Significant positively selective codons have been calculated using OmegaMap (Wilson and McVean 2006) and are marked with an X; the numbers represent the amino acid position in the MHC class II molecule

	dN	dS	dN/dS	(Z)	P	Positively selected codons in OmegaMap											
All gradient						11	13	27	28	34	37	47	60	63	70	74	78
PBS	0.159	0.040	3.975	3.894	0.000												
Non PBS	0.013	0.023	0.565	0.918	0.354												
All	0.043	0.027	1.592	1.33	0.183												
Northern cluster																	
PBS	0.066	0.013	5.076	2.672	0.004		**X**				**X**	**X**	**X**	**X**		**X**	
Non PBS	0.007	0.005	1.400	0.314	0.485												
All	0.019	0.006	3.166	2.144	0.017												
Southern cluster																	
PBS	0.170	0.047	3.617	3.621	0.000	**X**	**X**	**X**	**X**	**X**		**X**	**X**	**X**	**X**	**X**	**X**
Non PBS	0.014	0.028	0.5	0. 105	0.296												
All	0.047	0.032	1.468	1.075	0.285												
						0	0	1	0	3	0	0	1	2	0	0	0

Distance to Bondinas et al. 2007

**Fig. 2:**
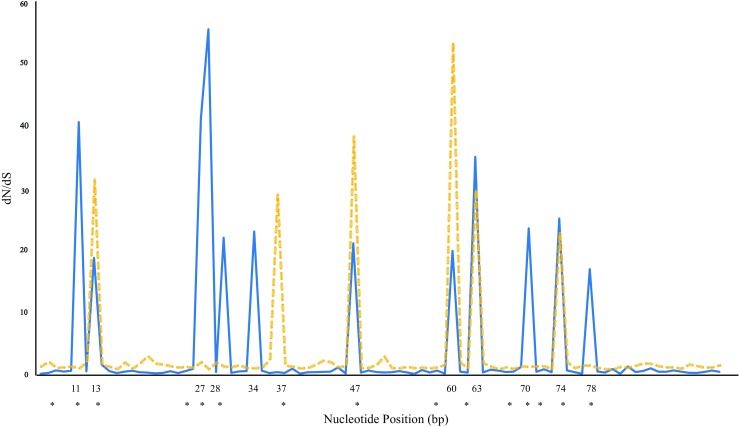
Sliding window dN/dS based on OmegaMap (Wilson and McVean 2006), for the 272-bp exon 2 fragments of the MHC II exon 2 (window size 5b, step size 15) in orange for the northern group and in blue for the southern group. PBR positions from Bondinas et al. (2007) are represented with a star below the x axes

A signature of natural selection was also detected using the dN/dS (*ω*) ratio and a signature of recombination (Rho; ρ) from the patterns of linkage disequilibrium by Bayesian interference in OmegaMap (Wilson and McVean 2006). As recommended, the model was run twice and then two runs were combined with “burn-in” of 50,000 iterations using the Summarize module provided by OmegaMap. In each simulation, we run 500,000 iterations and thinned every 1000 iterations to obtain the posterior distribution. We allowed for *ω* and *ρ* to vary across the codons as suggested by the manual, set the *ω* prior and *ρ* prior = inverse (0.010, 100), and *ω* and *ρ* model = independent. The remaining priors were set to improver inverse. In addition, we performed a sliding window analysis to visualize the position of the codons under selection (*ω* > 1) in MHC II exon 2 in concordance to OmegaMap results. All selection analyses were run separately for the northern and southern clusters. Analyses provided significant evidence of positive selection (*ω* > 1) in several positions (Table 2; Fig. 2 and Fig. S3). We found 11 codons in the southern cluster (positions 11, 13, 27, 28, 34, 47 60, 63, 70, 74, 78) and six codons in the northern cluster (positions 13, 37, 47, 60, 63, 74) showing signatures of positive selection with significant posterior probabilities (prob. of selection > 95%). Codons 11, 13, 27, 28, 37, 47, 70, 74, and 78 are part of the putative PBR of the MHC class II (Bondinas et al. 2007; marked in Table 2). While some of the codons exhibiting evidence of positive selection were not identified as part of the putative PBR (Bondinas et al. 2007), these codons were located close to the putative PBR sites (Table 2). Different signatures of natural selection between the northern and the southern clusters were also detected by the SELECTON server (Stern et al. 2007) and CodeML method (See supplementary Table S2). Both approaches are implemented within the Phylogenetic Analysis by Maximum Likelihood software package (PAML, version 4.9d) (Yang 2007). We followed a conservative criterion to select the codons identified at least by two methods to consider them as positive selective codons (Supplementary Table S2; see, e.g., Wlasiuk and Nachman 2010). All the codons selected for positive selection in OmegaMap were also highlighted in the two other methods. According to OmegaMap, there was no evidence of recombination in the MHC class II sequences (lower 95% CI *ρ* > 1). Moreover, as the power to detect recombination varies with different detection methods, we used an additional array of six methods from the RDP package (Martin et al. 2010) to analyze recombination (Supplementary Table S3). These revealed very weak indications of recombination, as four methods did not find recombination signals at all and two methods revealed a single recombination event in the south and the entire gradient.

Based on earlier studies on microsatellites and mitochondrial markers indicating dual recolonization routes to Scandinavia from southeastern Europe glacial refugia (Babik et al. 2004; Cortázar-Chinarro et al. 2017; Knopp and Merilä 2009), we assigned *R. arvalis* populations to two different clusters: a northern cluster consisting of populations from Umeå and Luleå and a southern cluster with populations from Germany, Skåne, and Uppsala. Consistent with this hypothesis, both the phylogenetic tree and the unrooted phylogenetic network in this study illustrated contrasting evolutionary histories in the northern and southern clusters, and we found striking differences in the signature of historical selection in the MHC II exon 2 between the northern and southern parts of the latitudinal gradient.

We found that almost all MHC II exon 2 haplotype sequences from the north clustered in a single clade, with the exception of the Raar_DAB*32, suggesting that they are more similar to each other than to southern alleles. On the contrary, alleles from the southern populations are scattered throughout the tree showing high allelic divergence (see Fig. 1). Alleles from northern and southern populations might have a different demographic origin, as predicted by the dual colonization hypothesis.

In terms of genetic diversity, we found that the MHC II exon 2 locus was more diverse in the southern cluster than in the northern in *R. arvalis*. As a result of post-glacial recolonization, genetically less diverse populations are often found at northern latitudes, differing from the southern populations due to their demographic history (Hewitt 1996, 1999; Taberlet et al. 1998). We found a total of 47 MHC II haplotypes in the south, 14 in the north and only four haplotypes which were shared between the north and south (Raar_DAB*18, Raar_DAB*22, Raar_DAB*9, Raar_DAB*15). The haplotype Raar_DAB*15 is present in Umeå but not further north in Luleå and in nine out of 12 populations along the gradient. It is a central haplotype in the network and connects the northern and southern clusters. This haplotype, very common in the southern cluster, could have arrived to the northern populations via introgression from the south across a hybrid zone. The average number of pairwise nucleotide differences (theta K) between the alleles is very high, indicating a high allelic divergence in the southern cluster (theta K = 11.7). It is considerably lower (theta K = 4.4) in the northern cluster, indicating that a majority of MHC II exon 2 alleles are very similar to each other in the north. This might suggest that MHC exon 2 is under uniform positive selection in the north, decreasing genetic variation.

Biotic interactions such as host-parasite interactions are believed to be of great importance for the origin and maintenance of genetic variation in latitudinal gradients. The number of parasite species increases towards the tropics, and there is evidence that parasites are more prevalent and virulent at low latitudes (Schemske et al. 2009). Smaller and more isolated *R. arvalis* populations at northern latitudes displaying a lower MHC II diversity might be more vulnerable to new emerging pathogens and, consequently, at higher risk of extinction as suggested in previous studies on mammals confronted with new pathogens due to global warming (e.g., Siddle et al. 2007). There is a need for further studies linking patterns of genetic variation and divergence with occurrence of infectious disease across latitudinal and other environmental gradients to better infer evolutionary processes shaping adaptive genetic variation in wildlife populations.

The high levels of polymorphism observed at most genes of the MHC are generated and maintained by means of balancing selection (Hughes and Nei 1988). In this study, we found deviating signals of positive selection at the MHC II exon 2 in the entire gradient and in the southern cluster compared to the northern cluster. While the high levels of genetic variation and allelic divergence in the southern cluster represent a classical pattern for MHC variation, the pattern of uniform positive selection in the north is deviating from this classical case and rather uncommon. Elevated and highly significant dN/dS ratios were found at the PBR at MHC II exon 2, though signals of historical selection in northern populations were weaker compared to those of southern populations. Consistent with previous studies (Bos and DeWoody 2005; Huang et al. 2016; Kiemnec-Tyburczy et al. 2012), we did not find any evidence that MHC II exon 2 locus has been undergoing recombination. Also, several positions along the MHC class II exon 2 showed evidence of positive selection, as revealed by OmegaMap. However, these positively selected positions deviated among the southern and the northern clusters. For the northern cluster, we found six codons under positive selection, whereas 11 codons were under positive selection for the southern cluster. This could mean that in spite of a strong signal of historical selection in the north, the effects of positive selection/drift could be acting on MHC and continue to act if the populations remain small and isolated, reflecting a differential selection patterns among northern and southern clusters.

Our earlier study (Cortázar-Chinarro et al. 2017) disentangling contemporary evolutionary forces acting on MHC II exon 2 along the same latitudinal gradient suggested that the MHC II exon 2 shows signs of contemporary diversifying selection in the southern populations but drift and uniform selection are dominant forces in the northern populations. In a similar example, a study on two mouse lemur species showed contrasting selection patterns acting on MHC II exon 2 locus, providing strong evidence of historical selection in *Microcebus murinus*, while selection was more relaxed for *M. berthae* (Pechouskova et al. 2015). The different selection patterns found in our study could be mediated by differential pathogen-driven selection processes together with the effects of the effective population size and the demographic history along the gradient. Although our results support differential selection patterns found in the previous studies on MHC II exon 2 variation (Cortázar-Chinarro et al. 2017; Pechouskova et al. 2015), we cannot rule out the possibility that our results may be partly due to lower sample sizes and fewer population contrasts in the north. Consequently, there is a need for additional studies with stronger sampling effort to further our understanding how genetic variation in *R. arvalis* is geographically partitioned and distributed from the Balkans to the northern part of Finland.

The results of the current study suggest that selection patterns differ from northern and southern clusters in *R. arvalis*. The MHC class II exon 2 has fewer sites subject to positive selection in the northern populations compared to the southern populations. We believe that the differential selection patterns observed in our study could be explained by the historical post-glacial recolonization processes and by the different selective pressures from parasites in the different populations along the present gradient and/or lower parasite diversity at northern latitudes, which should be investigated in more detail in future studies.

## Electronic supplementary material

Figure S1 Neighbor-joining tree for *Rana arvalis* MHC II exon 2 representing the 57 different alleles. Alleles present in the north are marked with an orange triangle and shared alleles between the northern and southern cluster are represented with a green rhombus. A natterjack toad sequence [Genbank HQ388291.1] from MHC II exon 2 was used as an outgroup. The scale bar represents substitution per site. (PDF 949 KB)

Figure S2 Haplotype network for the 57 MHC II exon 2 sequences. Every colored circle represents a different haplotype, the dots represent single nucleotide changes. Circles in blue represents haplotypes from the southern cluster, circles in orange represents haplotypes from the northern cluster and green circles represent shared haplotypes between the northern and southern cluster. (PDF 938 KB)

Figure S3 Aligment of amino acid sequences. PBR position from Bondinas et al. (2007) are marked with a +. The shaded amino acids are the OmegaMap (Wilson and McVean 2006) derived positively selected codon positions, a) in yellow for the northern cluster, b) and in blue for the southern cluster. (PDF 3.20 MB)

Table S1 (PDF 104 kb)

Table S2 (PDF 29.1 kb)

Table S3 (PDF 89.1 kb)
