# Supplementary material for: Signatures of historical selection on MHC reveal different selection patterns in the moor frog (Rana arvalis)
Source: Immunogenetics. 2018 Feb 1;70(7):477–84. doi: 10.1007/s00251-017-1051-1 (PMC6006221; doi:10.1007/s00251-017-1051-1)

Figure S1. Neighbour-joining tree for *Rana arvalis* MHC II exon 2 representing the 57 different alleles. Alleles present in the north are marked with an orange triangle and shared alleles between the northern and southern cluster are represented with a green rhombus. A natterjack toad sequence [Genbank HQ388291.1] from MHC II exon 2 was used as an outgroup. The scale bar represents substitution per site.

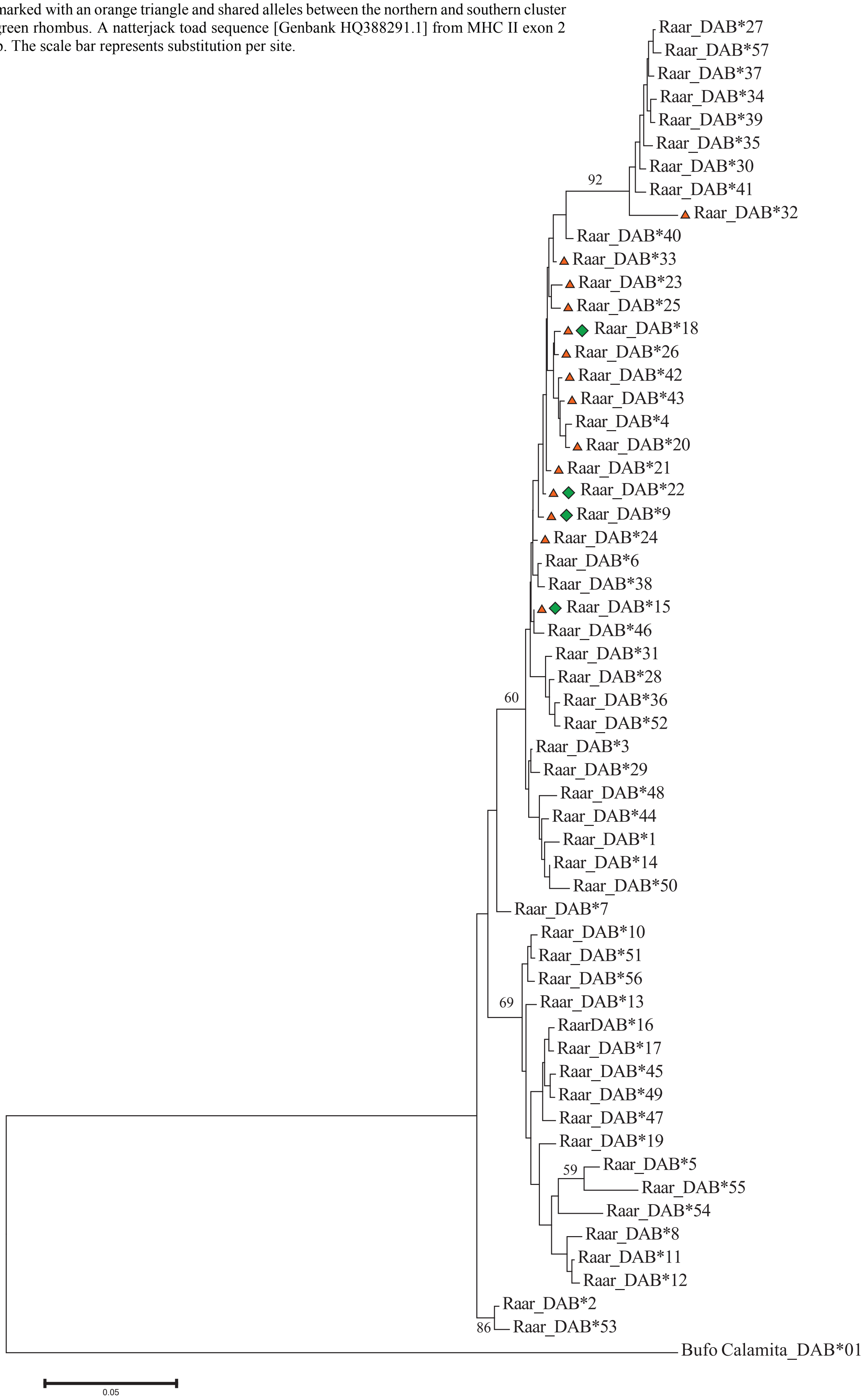

Supplement: Supplementary file 1 — Neighbor-joining tree for Rana arvalis MHC II exon 2 representing the 57 different alleles. Alleles present in the north are marked with an orange triangle and shared alleles between the northern and southern cluster are represented with a green rhombus. A natterjack toad sequence [Genbank HQ388291.1] from MHC II exon 2 was used as an outgroup. The scale bar represents substitution per site. (PDF 949 KB) [file 251_2017_1051_MOESM1_ESM.pdf]
