# Supplementary material for: Signatures of historical selection on MHC reveal different selection patterns in the moor frog (Rana arvalis)
Source: Immunogenetics. 2018 Feb 1;70(7):477–84. doi: 10.1007/s00251-017-1051-1 (PMC6006221; doi:10.1007/s00251-017-1051-1)

Figure S2. Haplotype network for the 57 MHC II exon 2 sequences. Every colored circle represents a different haplotype, the dots represent single nucleotide changes. Circles in blue represents haplotypes from the southern cluster, circles in orange represents haplotypes from the northern cluster and green circles represent shared haplotypes between the northern and southern cluster.

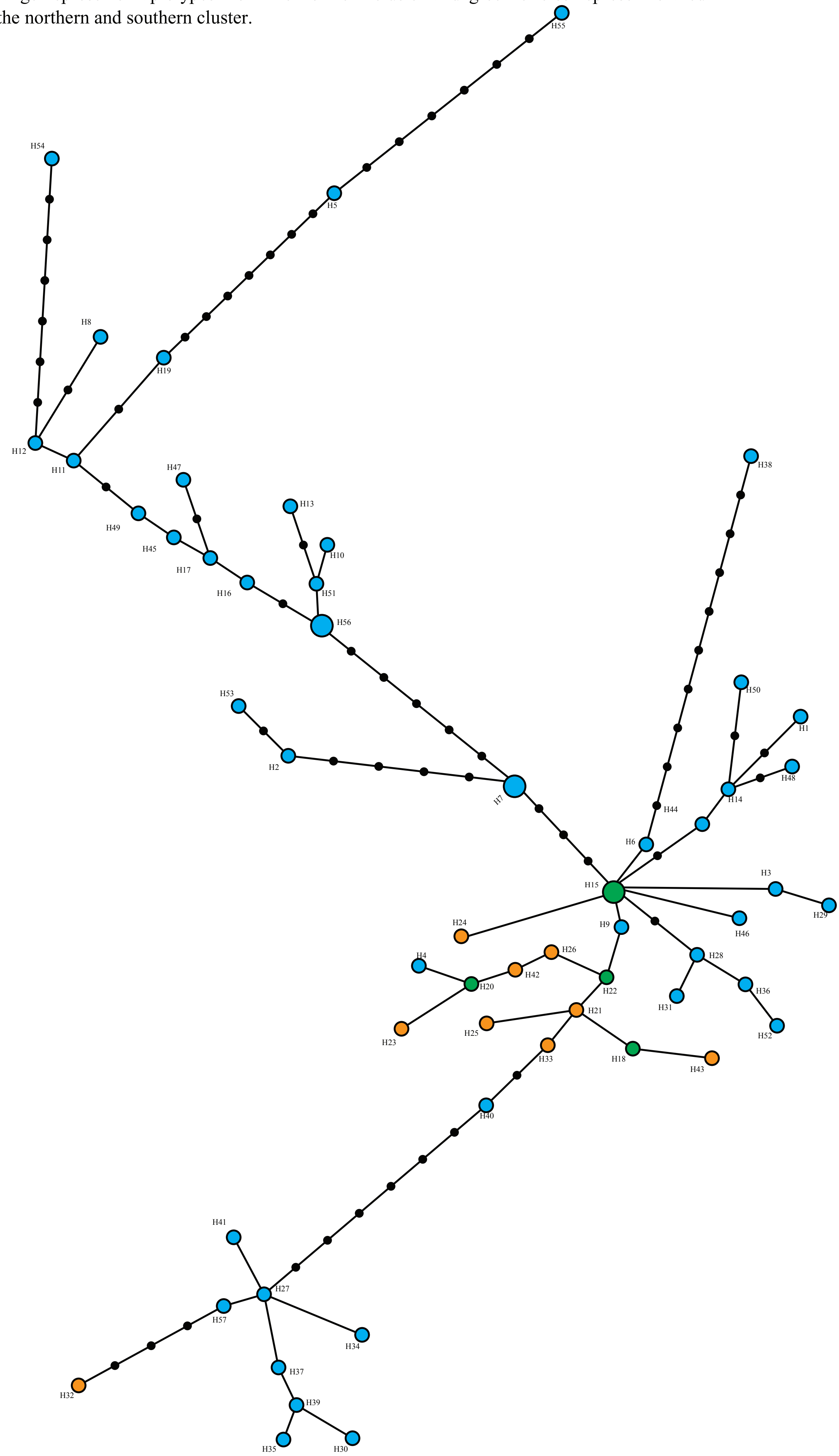

Supplement: Supplementary file 2 — Haplotype network for the 57 MHC II exon 2 sequences. Every colored circle represents a different haplotype, the dots represent single nucleotide changes. Circles in blue represents haplotypes from the southern cluster, circles in orange represents haplotypes from the northern cluster and green circles represent shared haplotypes between the northern and southern cluster. (PDF 938 KB) [file 251_2017_1051_MOESM2_ESM.pdf]
