# Supplementary material for: Signatures of historical selection on MHC reveal different selection patterns in the moor frog (Rana arvalis)
Source: Immunogenetics. 2018 Feb 1;70(7):477–84. doi: 10.1007/s00251-017-1051-1 (PMC6006221; doi:10.1007/s00251-017-1051-1)
Supplement: Supplementary file 3 — Aligment of amino acid sequences. PBR position from Bondinas et al. (2007) are marked with a +. The shaded amino acids are the OmegaMap (Wilson and McVean 2006) derived positively selected codon positions, a) in yellow for the northern cluster, b) and in blue for the southern cluster. (PDF 3.20 MB) [file 251_2017_1051_MOESM3_ESM.pdf]

Figure S3. Aligment of amino acid sequences. PBR position from Bondinas et al. (2007) are marked with a +. The shaded amino acids are the OmegaMap (Wilson and McVean 2006) derived positively selected codon positions, a) in yellow for the northern cluster, b) and in blue for the southern cluster.

Allele		6	7	8	9	10	11	12	13	14	15	16	17	18	19	20	21	22	23	24	25	26	27	28	29	30	31	32	33	34	35	36	37	38	39	40	41	42	43	44	45	46	47	48	49	50	51	52	53	54	55	56	57	58	59	60	61	62	63	64	65	66	67	68	69	70	71	72	73	74	75	76	77	78	79	80	81	82	83	84	85	86	87	88	89	90	91	92	93	94	95
					+		+		+													+		+		+							+										+										+				+						+			+	+			+				+							+	+			+	+					
Raar_DAB*01	-	D	F	V	Y	Q	D	K	G	Q	C	Y	Y	R	N	G	T	E	D	I	R	F	M	W	R	Y	I	Y	N	Q	E	E	F	V	Y	F	D	S	D	K	G	F	Y	I	P	R	T	E	Y	G	R	P	D	A	D	S	W	N	N	N	P	D	I	L	G	Q	A	R	A	V	V	E	T	V	C	K	H	N	Y	Q	T	Y	K	P	A	A	I	D	L	K	C
Raar_DAB*02	-	.	.	.	.	.	C	.	A	.	.	.	.	.	.	.	.	.	.	.	.	.	L	A	.	.	.	.	.	.	.	.	.	.	.	.	.	.	.	.	.	.	.	.	.	.	.	.	.	.	.	.	S	.	.	Y	.	.	S	D	.	.	.	.	.	.	.	.	.	.	.	.	.	.	.	.	.	.	.	.	.	.	.	.	.	.	.	.	.	.	.
Raar_DAB*03	-	.	.	.	.	.	C	.	.	.	.	.	.	.	.	.	.	.	.	.	.	.	.	.	.	.	.	.	.	.	.	.	.	.	.	.	.	.	.	.	.	.	.	.	.	.	.	.	.	.	.	.	.	.	.	.	.	.	.	.	.	.	.	.	.	.	.	.	.	.	.	.	.	.	.	.	.	.	.	.	.	.	.	.	.	.	.	.	.	.	.
Raar_DAB*04	-	.	.	.	.	.	C	.	.	.	.	.	.	.	.	.	.	.	.	.	.	.	.	.	.	.	.	.	.	.	.	.	Y	.	.	.	.	.	.	.	.	.	F	.	.	.	.	.	.	.	.	.	.	.	.	Y	.	.	S	.	.	.	.	.	.	.	.	.	.	A	.	.	.	.	.	.	.	.	.	.	.	.	.	.	.	.	.	.	.	.	.
Raar_DAB*05	-	.	.	.	.	.	N	.	.	.	.	.	.	.	.	.	.	.	.	.	.	.	L	D	.	H	.	.	.	.	.	.	.	.	.	.	.	.	.	.	.	.	F	.	.	.	.	.	.	.	.	.	.	.	.	.	.	.	S	D	.	.	.	.	.	R	K	.	.	E	.	.	.	.	.	.	.	.	.	.	.	.	.	.	.	.	.	.	.	.	.
Raar_DAB*06	-	.	.	.	.	.	C	.	A	.	.	.	.	.	.	.	.	.	.	.	.	.	.	.	.	.	.	.	.	.	.	.	.	.	.	.	.	.	.	.	.	.	.	.	.	.	.	.	.	.	.	.	.	.	.	Y	.	.	.	.	.	.	.	.	.	.	.	.	.	.	.	.	.	.	.	.	.	.	.	.	.	.	.	.	.	.	.	.	.	.	.
Raar_DAB*07	-	.	.	.	.	.	C	.	A	.	.	.	.	.	.	.	.	.	.	.	.	.	L	A	.	.	.	.	.	.	.	.	.	.	.	.	.	.	.	.	.	.	.	.	.	.	.	.	.	.	.	.	.	.	.	.	.	.	.	.	.	.	.	.	.	.	.	.	.	.	.	.	.	.	.	.	.	.	.	.	.	.	.	.	.	.	.	.	.	.	.
Raar_DAB*08	-	.	.	.	.	.	N	.	.	.	.	.	.	.	.	.	.	.	.	.	.	.	L	D	.	H	.	.	.	.	.	.	Y	.	.	.	.	.	.	.	.	.	F	.	.	.	.	.	.	.	.	.	.	.	.	.	.	.	S	.	.	.	.	.	.	R	.	.	.	E	.	.	.	L	.	.	.	.	.	.	.	.	.	.	.	.	.	.	.	.	.
Raar_DAB*09	-	.	.	.	.	.	C	.	A	.	.	.	.	.	.	.	.	.	.	.	.	.	.	.	.	.	.	.	.	.	.	.	.	.	.	.	.	.	.	.	.	.	F	.	.	.	.	.	.	.	.	.	.	.	.	.	.	.	.	.	.	.	.	.	.	.	.	.	.	.	.	.	.	.	.	.	.	.	.	.	.	.	.	.	.	.	.	.	.	.	.
Raar_DAB*10	-	.	.	.	.	.	N	.	.	.	.	.	.	.	.	.	.	.	.	.	.	.	L	D	.	H	.	.	.	R	.	.	.	.	.	.	.	.	.	.	.	.	F	.	.	.	.	.	.	.	.	.	.	.	.	.	.	.	.	.	.	.	.	.	.	.	.	.	.	.	.	.	.	.	.	.	.	.	.	.	.	.	.	.	.	.	.	.	.	.	.
Raar_DAB*11	-	.	.	.	.	.	N	.	.	.	.	.	.	.	.	.	.	.	.	.	.	.	L	D	.	H	.	.	.	R	.	.	.	.	.	.	.	.	.	.	.	.	F	.	.	.	.	.	.	.	.	.	.	.	.	.	.	.	S	.	.	.	.	.	.	R	.	.	.	.	.	.	.	L	.	.	.	.	.	.	.	.	.	.	.	.	.	.	.	.	.
Raar_DAB*12	-	.	.	.	.	.	N	.	.	.	.	.	.	.	.	.	.	.	.	.	.	.	L	D	.	H	.	.	.	R	.	.	.	.	.	.	.	.	.	.	.	.	F	.	.	.	.	.	.	.	.	.	.	.	.	.	.	.	S	.	.	.	.	.	.	R	.	.	.	E	.	.	.	L	.	.	.	.	.	.	.	.	.	.	.	.	.	.	.	.	.
Raar_DAB*13	-	.	.	.	.	.	N	.	.	.	.	.	.	.	.	.	.	.	.	.	.	.	L	D	.	H	.	.	.	R	.	.	.	.	.	.	.	.	.	.	.	.	.	.	.	.	.	.	.	.	.	.	.	.	.	Y	.	.	S	.	.	.	.	.	.	.	.	.	.	.	.	.	.	.	.	.	.	.	.	.	.	.	.	.	.	.	.	.	.	.	.
Raar_DAB*14	-	.	.	.	.	.	N	.	.	.	.	.	.	.	.	.	.	.	.	.	.	.	.	.	.	.	.	.	.	.	.	.	.	.	.	.	.	.	.	.	.	.	.	.	.	.	.	.	.	.	.	.	.	.	.	.	.	.	.	.	.	.	.	.	.	.	.	.	.	.	.	.	.	.	.	.	.	.	.	.	.	.	.	.	.	.	.	.	.	.	.
Raar_DAB*15	-	.	.	.	.	.	C	.	A	.	.	.	.	.	.	.	.	.	.	.	.	.	.	.	.	.	.	.	.	.	.	.	.	.	.	.	.	.	.	.	.	.	.	.	.	.	.	.	.	.	.	.	.	.	.	.	.	.	.	.	.	.	.	.	.	.	.	.	.	.	.	.	.	.	.	.	.	.	.	.	.	.	.	.	.	.	.	.	.	.	.
Raar_DAB*16	-	.	.	.	.	.	N	.	A	.	.	.	.	.	.	.	.	.	.	.	.	.	L	D	.	H	.	.	.	R	.	.	.	.	.	.	.	.	.	.	.	.	.	.	.	.	.	.	.	.	.	.	.	.	.	.	.	.	.	.	.	.	.	.	.	.	.	.	.	.	.	.	.	L	.	.	.	.	.	.	.	.	.	.	.	.	.	.	.	.	.
Raar_DAB*17	-	.	.	.	.	.	N	.	.	.	.	.	.	.	.	.	.	.	.	.	.	.	L	D	.	H	.	.	.	R	.	.	.	.	.	.	.	.	.	.	.	.	.	.	.	.	.	.	.	.	.	.	.	.	.	.	.	.	.	.	.	.	.	.	.	.	.	.	.	.	.	.	.	L	.	.	.	.	.	.	.	.	.	.	.	.	.	.	.	.	.
Raar_DAB*18	-	.	.	.	.	.	C	.	A	.	.	.	.	.	.	.	.	.	.	.	.	.	.	.	.	.	.	.	.	.	.	.	Y	.	.	.	.	.	.	.	.	.	F	.	.	.	.	.	.	.	.	.	.	.	.	Y	.	.	S	.	.	.	.	.	.	.	.	.	.	.	.	.	.	.	.	.	.	.	.	.	.	.	.	.	.	.	.	.	.	.	.
Raar_DAB*19	-	.	.	.	.	.	N	.	.	.	.	.	.	.	.	.	.	.	.	.	.	.	L	D	.	H	.	.	.	R	.	.	.	.	.	.	.	.	.	.	.	.	F	.	.	.	.	.	.	.	.	.	.	.	.	.	.	.	S	.	.	.	.	.	.	R	.	.	.	.	.	.	.	.	.	.	.	.	.	.	.	.	.	.	.	.	.	.	.	.	.
Raar_DAB*20	-	.	.	.	.	.	C	.	.	.	.	.	.	.	.	.	.	.	.	.	.	.	.	.	.	.	.	.	.	.	.	.	Y	.	.	.	.	.	.	.	.	.	F	.	.	.	.	.	.	.	.	.	.	.	.	.	.	.	S	.	.	.	.	.	.	.	.	.	.	A	.	.	.	.	.	.	.	.	.	.	.	.	.	.	.	.	.	.	.	.	.
Raar_DAB*21	-	.	.	.	.	.	C	.	A	.	.	.	.	.	.	.	.	.	.	.	.	.	.	.	.	.	.	.	.	.	.	.	.	.	.	.	.	.	.	.	.	.	F	.	.	.	.	.	.	.	.	.	.	.	.	Y	.	.	S	.	.	.	.	.	.	.	.	.	.	.	.	.	.	.	.	.	.	.	.	.	.	.	.	.	.	.	.	.	.	.	.
Raar_DAB*22	-	.	.	.	.	.	C	.	A	.	.	.	.	.	.	.	.	.	.	.	.	.	.	.	.	.	.	.	.	.	.	.	.	.	.	.	.	.	.	.	.	.	F	.	.	.	.	.	.	.	.	.	.	.	.	.	.	.	S	.	.	.	.	.	.	.	.	.	.	.	.	.	.	.	.	.	.	.	.	.	.	.	.	.	.	.	.	.	.	.	.
Raar_DAB*23	-	.	.	.	.	.	C	.	.	.	.	.	.	.	.	.	.	.	.	.	.	.	.	.	.	.	.	.	.	.	.	.	.	.	.	.	.	.	.	.	.	.	F	.	.	.	.	.	.	.	.	.	.	.	.	.	.	.	S	.	.	.	.	.	.	.	.	.	.	A	.	.	.	.	.	.	.	.	.	.	.	.	.	.	.	.	.	.	.	.	.
Raar_DAB*24	-	.	.	.	.	.	C	.	A	.	.	.	.	.	.	.	.	.	.	.	.	.	.	.	.	.	.	.	.	.	.	.	.	.	.	.	.	.	.	.	.	.	.	.	.	.	.	.	.	.	.	.	.	.	.	.	.	.	S	.	.	.	.	.	.	.	.	.	.	.	.	.	.	.	.	.	.	.	.	.	.	.	.	.	.	.	.	.	.	.	.
Raar_DAB*25	-	.	.	.	.	.	C	.	A	.	.	.	.	.	.	.	.	.	.	.	.	.	.	.	.	.	.	.	.	.	.	.	.	.	.	.	.	.	.	.	.	.	F	.	.	.	.	.	.	.	.	.	.	.	.	Y	.	.	S	.	.	.	.	.	.	.	.	.	.	A	.	.	.	.	.	.	.	.	.	.	.	.	.	.	.	.	.	.	.	.	.
Raar_DAB*26	-	.	.	.	.	.	C	.	A	.	.	.	.	.	.	.	.	.	.	.	.	.	.	.	.	.	.	.	.	.	.	.	Y	.	.	.	.	.	.	.	.	.	F	.	.	.	.	.	.	.	.	.	.	.	.	.	.	.	S	.	.	.	.	.	.	.	.	.	.	.	.	.	.	.	.	.	.	.	.	.	.	.	.	.	.	.	.	.	.	.	.
Raar_DAB*27	-	.	.	.	.	.	G	.	.	.	.	.	.	.	.	.	.	.	.	.	.	.	.	.	.	.	.	.	.	.	.	.	L	.	.	.	.	.	.	.	.	.	F	.	.	.	.	.	.	.	.	.	.	.	.	Y	.	.	S	.	.	.	.	.	.	R	F	.	.	E	.	.	.	.	.	.	.	.	.	.	.	.	.	.	.	.	.	.	.	.	.
Raar_DAB*28	-	.	.	.	.	.	C	.	A	.	.	.	.	.	.	.	.	.	.	.	.	.	.	.	.	.	.	.	.	.	.	.	.	.	.	.	.	.	.	.	.	.	.	.	.	.	.	.	.	.	.	.	S	.	.	.	.	.	.	.	.	.	.	.	.	.	.	.	.	.	.	.	.	.	.	.	.	.	.	.	.	.	.	.	.	.	.	.	.	.	.
Raar_DAB*29	-	.	.	.	.	.	G	.	.	.	.	.	.	.	.	.	.	.	.	.	.	.	.	.	.	.	.	.	.	.	.	.	.	.	.	.	.	.	.	.	.	.	.	.	.	.	.	.	.	.	.	.	.	.	.	.	.	.	.	.	.	.	.	.	.	.	.	.	.	.	.	.	.	.	.	.	.	.	.	.	.	.	.	.	.	.	.	.	.	.	.
Raar_DAB*30	-	.	.	.	.	.	G	.	A	.	.	.	.	.	.	.	.	.	.	.	.	.	.	.	.	.	.	.	.	.	.	.	.	.	.	.	.	.	.	.	.	.	.	.	.	.	.	.	.	.	.	.	.	.	.	Y	.	.	S	.	.	.	.	.	.	R	F	.	.	E	.	.	.	.	.	.	.	.	.	.	.	.	.	.	.	.	.	.	.	.	.
Raar_DAB*31	-	.	.	.	.	.	C	.	A	.	.	.	.	.	.	.	.	.	.	.	.	.	.	.	.	.	.	.	.	.	.	.	.	.	.	.	.	.	.	.	.	.	.	.	.	.	.	.	.	.	.	.	S	.	.	.	.	.	S	.	.	.	.	.	.	.	.	.	.	.	.	.	.	.	.	.	.	.	.	.	.	.	.	.	.	.	.	.	.	.	.
Raar_DAB*32	-	.	.	.	.	.	G	.	.	.	.	.	.	.	.	.	.	.	.	.	.	.	L	S	.	.	.	.	.	R	.	.	L	.	.	.	.	.	.	.	.	.	F	.	.	.	.	.	.	.	.	.	.	.	.	Y	.	.	S	.	.	.	.	.	.	R	F	.	.	E	.	.	.	.	.	.	.	.	.	.	.	.	.	.	.	.	.	.	.	.	.
Raar_DAB*33	-	.	.	.	.	.	C	.	.	.	.	.	.	.	.	.	.	.	.	.	.	.	.	.	.	.	.	.	.	.	.	.	.	.	.	.	.	.	.	.	.	.	F	.	.	.	.	.	.	.	.	.	.	.	.	Y	.	.	S	.	.	.	.	.	.	.	.	.	.	.	.	.	.	.	.	.	.	.	.	.	.	.	.	.	.	.	.	.	.	.	.
Raar_DAB*34	-	.	.	.	.	.	G	.	A	.	.	.	.	.	.	.	.	.	.	.	.	.	.	.	.	.	.	.	.	.	.	.	L	.	.	.	.	.	.	.	.	.	F	.	.	.	.	.	.	.	.	.	.	.	.	Y	.	.	S	.	.	.	.	.	.	R	F	.	.	E	.	.	.	.	.	.	.	.	.	.	.	.	.	.	.	.	.	.	.	.	.
Raar_DAB*35	-	.	.	.	.	.	C	.	A	.	.	.	.	.	.	.	.	.	.	.	.	.	.	.	.	.	.	.	.	.	.	.	L	.	.	.	.	.	.	.	.	.	.	.	.	.	.	.	.	.	.	.	.	.	.	Y	.	.	S	.	.	.	.	.	.	R	F	.	.	E	.	.	.	.	.	.	.	.	.	.	.	.	.	.	.	.	.	.	.	.	.
Raar_DAB*36	-	.	.	.	.	.	G	.	A	.	.	.	.	.	.	.	.	.	.	.	.	.	.	.	.	.	.	.	.	.	.	.	.	.	.	.	.	.	.	.	.	.	.	.	.	.	.	.	.	.	.	.	S	.	.	.	.	.	.	.	.	.	.	.	.	.	.	.	.	.	.	.	.	.	.	.	.	.	.	.	.	.	.	.	.	.	.	.	.	.	.
Raar_DAB*37	-	.	.	.	.	.	G	.	.	.	.	.	.	.	.	.	.	.	.	.	.	.	.	.	.	.	.	.	.	.	.	.	L	.	.	.	.	.	.	.	.	.	.	.	.	.	.	.	.	.	.	.	.	.	.	Y	.	.	S	.	.	.	.	.	.	R	F	.	.	E	.	.	.	.	.	.	.	.	.	.	.	.	.	.	.	.	.	.	.	.	.
Raar_DAB*38	-	.	.	.	.	.	C	.	A	.	.	.	.	.	.	.	.	.	.	.	.	.	.	.	.	.	.	.	.	.	.	.	.	.	.	.	.	.	.	.	.	.	.	.	.	.	.	.	.	.	.	.	.	.	.	Y	.	.	S	.	.	.	.	.	.	.	.	.	.	.	.	.	.	.	.	.	.	.	.	.	.	.	.	.	.	.	.	.	.	.	.
Raar_DAB*39	-	.	.	.	.	.	G	.	A	.	.	.	.	.	.	.	.	.	.	.	.	.	.	.	.	.	.	.	.	.	.	.	L	.	.	.	.	.	.	.	.	.	.	.	.	.	.	.	.	.	.	.	.	.	.	Y	.	.	S	.	.	.	.	.	.	R	F	.	.	E	.	.	.	.	.	.	.	.	.	.	.	.	.	.	.	.	.	.	.	.	.
Raar_DAB*40	-	.	.	.	.	.	G	.	.	.	.	.	.	.	.	.	.	.	.	.	.	.	.	.	.	.	.	.	.	.	.	.	L	.	.	.	.	.	.	.	.	.	F	.	.	.	.	.	.	.	.	.	.	.	.	Y	.	.	S	.	.	.	.	.	.	.	.	.	.	.	.	.	.	.	.	.	.	.	.	.	.	.	.	.	.	.	.	.	.	.	.
Raar_DAB*41	-	.	.	.	.	.	G	.	.	.	.	.	.	.	.	.	.	.	.	.	.	.	.	.	.	.	.	.	.	.	.	.	.	.	.	.	.	.	.	.	.	.	F	.	.	.	.	.	.	.	.	.	.	.	.	Y	.	.	S	.	.	.	.	.	.	R	F	.	.	E	.	.	.	.	.	.	.	.	.	.	.	.	.	.	.	.	.	.	.	.	.
Raar_DAB*42	-	.	.	.	.	.	C	.	.	.	.	.	.	.	.	.	.	.	.	.	.	.	.	.	.	.	.	.	.	.	.	.	Y	.	.	.	.	.	.	.	.	.	F	.	.	.	.	.	.	.	.	.	.	.	.	.	.	.	S	.	.	.	.	.	.	.	.	.	.	.	.	.	.	.	.	.	.	.	.	.	.	.	.	.	.	.	.	.	.	.	.
Raar_DAB*43	-	.	.	.	.	.	C	.	.	.	.	.	.	.	.	.	.	.	.	.	.	.	.	.	.	.	.	.	.	.	.	.	Y	.	.	.	.	.	.	.	.	.	F	.	.	.	.	.	.	.	.	.	.	.	.	Y	.	.	S	.	.	.	.	.	.	.	.	.	.	.	.	.	.	.	.	.	.	.	.	.	.	.	.	.	.	.	.	.	.	.	.
Raar_DAB*44	-	.	.	.	.	.	N	.	A	.	.	.	.	.	.	.	.	.	.	.	.	.	.	.	.	.	.	.	.	.	.	.	.	.	.	.	.	.	.	.	.	.	.	.	.	.	.	.	.	.	.	.	.	.	.	.	.	.	.	.	.	.	.	.	.	.	.	.	.	.	.	.	.	.	.	.	.	.	.	.	.	.	.	.	.	.	.	.	.	.	.
Raar_DAB*45	-	.	.	.	.	.	N	.	.	.	.	.	.	.	.	.	.	.	.	.	.	.	L	D	.	H	.	.	.	R	.	.	.	.	.	.	.	.	.	.	.	.	F	.	.	.	.	.	.	.	.	.	.	.	.	.	.	.	.	.	.	.	.	.	.	.	.	.	.	.	.	.	.	L	.	.	.	.	.	.	.	.	.	.	.	.	.	.	.	.	.
Raar_DAB*46	-	.	.	.	.	.	C	.	A	.	.	.	.	.	.	.	.	.	.	.	.	.	.	.	.	.	.	.	.	.	.	.	.	.	.	.	.	.	.	.	.	.	.	.	.	.	.	.	.	.	.	.	.	.	.	.	.	.	.	.	.	.	.	.	.	.	.	.	.	.	.	.	.	.	.	.	.	.	.	.	S	.	.	.	.	.	.	.	.	.
Raar_DAB*47	-	.	.	.	.	.	N	.	.	.	.	.	.	.	.	.	.	.	.	.	.	.	L	D	.	H	.	.	.	R	.	.	.	.	.	.	.	.	.	.	.	.	.	.	.	.	.	.	.	.	.	.	.	.	.	Y	.	.	S	.	.	.	.	.	.	.	.	.	.	.	.	.	.	L	.	.	.	.	.	.	.	.	.	.	.	.	.	.	.	.	.
Raar_DAB*48	-	.	.	.	.	.	N	.	.	.	.	.	.	.	.	.	.	.	.	.	.	.	.	.	.	.	.	.	.	.	.	.	.	.	.	.	.	.	.	.	.	.	.	.	.	.	.	.	.	.	.	.	.	.	.	.	.	.	S	.	.	.	.	.	.	.	.	.	.	.	.	.	.	.	.	.	.	.	.	.	.	.	.	.	.	.	.	.	.	.	.
Raar_DAB*49	-	.	.	.	.	.	N	.	.	.	.	.	.	.	.	.	.	.	.	.	.	.	L	D	.	H	.	.	.	R	.	.	.	.	.	.	.	.	.	.	.	.	F	.	.	.	.	.	.	.	.	.	.	.	.	.	.	.	S	.	.	.	.	.	.	.	.	.	.	.	.	.	.	L	.	.	.	.	.	.	.	.	.	.	.	.	.	.	.	.	.
Raar_DAB*50	-	.	.	.	.	.	N	.	.	.	.	.	.	.	.	.	.	.	.	.	.	.	.	.	.	.	.	.	.	.	.	.	.	.	.	.	.	.	.	.	.	.	.	.	.	.	.	.	.	.	.	.	.	.	.	.	.	.	.	.	.	.	.	.	.	.	.	.	.	.	.	.	.	L	.	.	.	.	.	.	.	.	.	.	.	.	.	.	.	.	.
Raar_DAB*51	-	.	.	.	.	.	N	.	.	.	.	.	.	.	.	.	.	.	.	.	.	.	L	D	.	H	.	.	.	R	.	.	.	.	.	.	.	.	.	.	.	.	.	.	.	.	.	.	.	.	.	.	.	.	.	.	.	.	.	.	.	.	.	.	.	.	.	.	.	.	.	.	.	.	.	.	.	.	.	.	.	.	.	.	.	.	.	.	.	.	.
Raar_DAB*52	-	.	.	.	.	.	G	.	.	.	.	.	.	.	.	.	.	.	.	.	.	.	.	.	.	.	.	.	.	.	.	.	.	.	.	.	.	.	.	.	.	.	.	.	.	.	.	.	.	.	.	.	S	.	.	.	.	.	.	.	.	.	.	.	.	.	.	.	.	.	.	.	.	.	.	.	.	.	.	.	.	.	.	.	.	.	.	.	.	.	.
Raar_DAB*53	-	.	.	.	.	.	N	.	A	.	.	.	.	.	.	.	.	.	.	.	.	.	L	A	.	.	.	.	.	.	.	.	.	.	.	.	.	.	.	.	.	.	.	.	.	.	.	.	.	.	.	.	S	.	.	Y	.	.	S	D	.	.	.	.	.	.	.	.	.	.	.	.	.	.	.	.	.	.	.	.	.	.	.	.	.	.	.	.	.	.	.
Raar_DAB*54	-	.	.	.	.	.	N	.	.	.	.	.	.	.	.	.	.	.	.	.	.	.	L	D	.	H	.	.	.	.	.	.	.	.	.	.	.	.	.	.	.	.	F	.	.	.	.	.	.	.	.	.	S	.	.	Y	.	.	S	D	.	.	.	.	.	.	.	.	.	E	.	.	.	L	.	.	.	.	.	.	.	.	.	.	.	.	.	.	.	.	.
Raar_DAB*55	-	.	.	.	.	.	Y	.	.	.	.	.	.	.	.	.	.	.	.	.	.	.	L	D	.	H	.	.	.	.	.	.	Y	.	.	.	.	.	.	.	.	.	F	.	.	.	.	.	.	.	.	.	S	.	.	Y	.	.	S	D	.	.	.	.	.	R	K	.	.	E	.	.	.	L	.	.	.	.	.	.	.	.	.	.	.	.	.	.	.	.	.
Raar_DAB*56	-	.	.	.	.	.	N	.	A	.	.	.	.	.	.	.	.	.	.	.	.	.	L	D	.	H	.	.	.	R	.	.	.	.	.	.	.	.	.	.	.	.	.	.	.	.	.	.	.	.	.	.	.	.	.	.	.	.	.	.	.	.	.	.	.	.	.	.	.	.	.	.	.	.	.	.	.	.	.	.	.	.	.	.	.	.	.	.	.	.	.
Raar_DAB*57	-	.	.	.	.	.	G	.	.	.	.	.	.	.	.	.	.	.	.	.	.	.	.	.	.	.	.	.	.	R	.	.	L	.	.	.	.	.	.	.	.	.	F	.	.	.	.	.	.	.	.	.	.	.	.	Y	.	.	S	.	.	.	.	.	.	R	F	.	.	E	.	.	.	.	.	.	.	.	.	.	.	.	.	.	.	.	.	.	.	.	.

		6	7	8	9	10	11	12	13	14	15	16	17	18	19	20	21	22	23	24	25	26	27	28	29	30	31	32	33	34	35	36	37	38	39	40	41	42	43	44	45	46	47	48	49	50	51	52	53	54	55	56	57	58	59	60	61	62	63	64	65	66	67	68	69	70	71	72	73	74	75	76	77	78	79	80	81	82	83	84	85	86	87	88	89	90	91	92	93	94	95
					+		+		+													+		+		+							+										+										+				+						+			+	+			+				+							+	+			+	+					
**Raar_DAB*01**	-	D	F	V	Y	Q	D	K	G	Q	C	Y	Y	R	N	G	T	E	D	I	R	F	M	W	R	Y	I	Y	N	Q	E	E	F	V	Y	F	D	S	D	K	G	F	Y	I	P	R	T	E	Y	G	R	P	D	A	D	S	W	N	N	N	P	D	I	L	G	Q	A	R	A	V	V	E	T	V	C	K	H	N	Y	Q	T	Y	K	P	A	A	I	D	L	K	C
**Raar_DAB*02**	-	.	.	.	.	.	C	.	A	.	.	.	.	.	.	.	.	.	.	.	.	.	L	A	.	.	.	.	.	.	.	.	.	.	.	.	.	.	.	.	.	.	.	.	.	.	.	.	.	.	.	.	S	.	.	Y	.	.	S	D	.	.	.	.	.	.	.	.	.	.	.	.	.	.	.	.	.	.	.	.	.	.	.	.	.	.	.	.	.	.	.
**Raar_DAB*03**	-	.	.	.	.	.	C	.	.	.	.	.	.	.	.	.	.	.	.	.	.	.	.	.	.	.	.	.	.	.	.	.	.	.	.	.	.	.	.	.	.	.	.	.	.	.	.	.	.	.	.	.	.	.	.	.	.	.	.	.	.	.	.	.	.	.	.	.	.	.	.	.	.	.	.	.	.	.	.	.	.	.	.	.	.	.	.	.	.	.	.
**Raar_DAB*04**	-	.	.	.	.	.	C	.	.	.	.	.	.	.	.	.	.	.	.	.	.	.	.	.	.	.	.	.	.	.	.	.	Y	.	.	.	.	.	.	.	.	.	F	.	.	.	.	.	.	.	.	.	.	.	.	Y	.	.	S	.	.	.	.	.	.	.	.	.	.	A	.	.	.	.	.	.	.	.	.	.	.	.	.	.	.	.	.	.	.	.	.
**Raar_DAB*05**	-	.	.	.	.	.	N	.	.	.	.	.	.	.	.	.	.	.	.	.	.	.	L	D	.	H	.	.	.	.	.	.	.	.	.	.	.	.	.	.	.	.	F	.	.	.	.	.	.	.	.	.	.	.	.	.	.	.	S	D	.	.	.	.	.	R	K	.	.	E	.	.	.	.	.	.	.	.	.	.	.	.	.	.	.	.	.	.	.	.	.
**Raar_DAB*06**	-	.	.	.	.	.	C	.	A	.	.	.	.	.	.	.	.	.	.	.	.	.	.	.	.	.	.	.	.	.	.	.	.	.	.	.	.	.	.	.	.	.	.	.	.	.	.	.	.	.	.	.	.	.	.	Y	.	.	.	.	.	.	.	.	.	.	.	.	.	.	.	.	.	.	.	.	.	.	.	.	.	.	.	.	.	.	.	.	.	.	.
**Raar_DAB*07**	-	.	.	.	.	.	C	.	A	.	.	.	.	.	.	.	.	.	.	.	.	.	L	A	.	.	.	.	.	.	.	.	.	.	.	.	.	.	.	.	.	.	.	.	.	.	.	.	.	.	.	.	.	.	.	.	.	.	.	.	.	.	.	.	.	.	.	.	.	.	.	.	.	.	.	.	.	.	.	.	.	.	.	.	.	.	.	.	.	.	.
**Raar_DAB*08**	-	.	.	.	.	.	N	.	.	.	.	.	.	.	.	.	.	.	.	.	.	.	L	D	.	H	.	.	.	.	.	.	Y	.	.	.	.	.	.	.	.	.	F	.	.	.	.	.	.	.	.	.	.	.	.	.	.	.	S	.	.	.	.	.	.	R	.	.	.	E	.	.	.	L	.	.	.	.	.	.	.	.	.	.	.	.	.	.	.	.	.
**Raar_DAB*09**	-	.	.	.	.	.	C	.	A	.	.	.	.	.	.	.	.	.	.	.	.	.	.	.	.	.	.	.	.	.	.	.	.	.	.	.	.	.	.	.	.	.	F	.	.	.	.	.	.	.	.	.	.	.	.	.	.	.	.	.	.	.	.	.	.	.	.	.	.	.	.	.	.	.	.	.	.	.	.	.	.	.	.	.	.	.	.	.	.	.	.
**Raar_DAB*10**	-	.	.	.	.	.	N	.	.	.	.	.	.	.	.	.	.	.	.	.	.	.	L	D	.	H	.	.	.	R	.	.	.	.	.	.	.	.	.	.	.	.	F	.	.	.	.	.	.	.	.	.	.	.	.	.	.	.	.	.	.	.	.	.	.	.	.	.	.	.	.	.	.	.	.	.	.	.	.	.	.	.	.	.	.	.	.	.	.	.	.
**Raar_DAB*11**	-	.	.	.	.	.	N	.	.	.	.	.	.	.	.	.	.	.	.	.	.	.	L	D	.	H	.	.	.	R	.	.	.	.	.	.	.	.	.	.	.	.	F	.	.	.	.	.	.	.	.	.	.	.	.	.	.	.	S	.	.	.	.	.	.	R	.	.	.	.	.	.	.	L	.	.	.	.	.	.	.	.	.	.	.	.	.	.	.	.	.
**Raar_DAB*12**	-	.	.	.	.	.	N	.	.	.	.	.	.	.	.	.	.	.	.	.	.	.	L	D	.	H	.	.	.	R	.	.	.	.	.	.	.	.	.	.	.	.	F	.	.	.	.	.	.	.	.	.	.	.	.	.	.	.	S	.	.	.	.	.	.	R	.	.	.	E	.	.	.	L	.	.	.	.	.	.	.	.	.	.	.	.	.	.	.	.	.
**Raar_DAB*13**	-	.	.	.	.	.	N	.	.	.	.	.	.	.	.	.	.	.	.	.	.	.	L	D	.	H	.	.	.	R	.	.	.	.	.	.	.	.	.	.	.	.	.	.	.	.	.	.	.	.	.	.	.	.	.	Y	.	.	S	.	.	.	.	.	.	.	.	.	.	.	.	.	.	.	.	.	.	.	.	.	.	.	.	.	.	.	.	.	.	.	.
**Raar_DAB*14**	-	.	.	.	.	.	N	.	.	.	.	.	.	.	.	.	.	.	.	.	.	.	.	.	.	.	.	.	.	.	.	.	.	.	.	.	.	.	.	.	.	.	.	.	.	.	.	.	.	.	.	.	.	.	.	.	.	.	.	.	.	.	.	.	.	.	.	.	.	.	.	.	.	.	.	.	.	.	.	.	.	.	.	.	.	.	.	.	.	.	.
**Raar_DAB*15**	-	.	.	.	.	.	C	.	A	.	.	.	.	.	.	.	.	.	.	.	.	.	.	.	.	.	.	.	.	.	.	.	.	.	.	.	.	.	.	.	.	.	.	.	.	.	.	.	.	.	.	.	.	.	.	.	.	.	.	.	.	.	.	.	.	.	.	.	.	.	.	.	.	.	.	.	.	.	.	.	.	.	.	.	.	.	.	.	.	.	.
**Raar_DAB*16**	-	.	.	.	.	.	N	.	A	.	.	.	.	.	.	.	.	.	.	.	.	.	L	D	.	H	.	.	.	R	.	.	.	.	.	.	.	.	.	.	.	.	.	.	.	.	.	.	.	.	.	.	.	.	.	.	.	.	.	.	.	.	.	.	.	.	.	.	.	.	.	.	.	L	.	.	.	.	.	.	.	.	.	.	.	.	.	.	.	.	.
**Raar_DAB*17**	-	.	.	.	.	.	N	.	.	.	.	.	.	.	.	.	.	.	.	.	.	.	L	D	.	H	.	.	.	R	.	.	.	.	.	.	.	.	.	.	.	.	.	.	.	.	.	.	.	.	.	.	.	.	.	.	.	.	.	.	.	.	.	.	.	.	.	.	.	.	.	.	.	L	.	.	.	.	.	.	.	.	.	.	.	.	.	.	.	.	.
**Raar_DAB*18**	-	.	.	.	.	.	C	.	A	.	.	.	.	.	.	.	.	.	.	.	.	.	.	.	.	.	.	.	.	.	.	.	Y	.	.	.	.	.	.	.	.	.	F	.	.	.	.	.	.	.	.	.	.	.	.	Y	.	.	S	.	.	.	.	.	.	.	.	.	.	.	.	.	.	.	.	.	.	.	.	.	.	.	.	.	.	.	.	.	.	.	.
**Raar_DAB*19**	-	.	.	.	.	.	N	.	.	.	.	.	.	.	.	.	.	.	.	.	.	.	L	D	.	H	.	.	.	R	.	.	.	.	.	.	.	.	.	.	.	.	F	.	.	.	.	.	.	.	.	.	.	.	.	.	.	.	S	.	.	.	.	.	.	R	.	.	.	.	.	.	.	.	.	.	.	.	.	.	.	.	.	.	.	.	.	.	.	.	.
**Raar_DAB*20**	-	.	.	.	.	.	C	.	.	.	.	.	.	.	.	.	.	.	.	.	.	.	.	.	.	.	.	.	.	.	.	.	Y	.	.	.	.	.	.	.	.	.	F	.	.	.	.	.	.	.	.	.	.	.	.	.	.	.	S	.	.	.	.	.	.	.	.	.	.	A	.	.	.	.	.	.	.	.	.	.	.	.	.	.	.	.	.	.	.	.	.
**Raar_DAB*21**	-	.	.	.	.	.	C	.	A	.	.	.	.	.	.	.	.	.	.	.	.	.	.	.	.	.	.	.	.	.	.	.	.	.	.	.	.	.	.	.	.	.	F	.	.	.	.	.	.	.	.	.	.	.	.	Y	.	.	S	.	.	.	.	.	.	.	.	.	.	.	.	.	.	.	.	.	.	.	.	.	.	.	.	.	.	.	.	.	.	.	.
**Raar_DAB*22**	-	.	.	.	.	.	C	.	A	.	.	.	.	.	.	.	.	.	.	.	.	.	.	.	.	.	.	.	.	.	.	.	.	.	.	.	.	.	.	.	.	.	F	.	.	.	.	.	.	.	.	.	.	.	.	.	.	.	S	.	.	.	.	.	.	.	.	.	.	.	.	.	.	.	.	.	.	.	.	.	.	.	.	.	.	.	.	.	.	.	.
**Raar_DAB*23**	-	.	.	.	.	.	C	.	.	.	.	.	.	.	.	.	.	.	.	.	.	.	.	.	.	.	.	.	.	.	.	.	.	.	.	.	.	.	.	.	.	.	F	.	.	.	.	.	.	.	.	.	.	.	.	.	.	.	S	.	.	.	.	.	.	.	.	.	.	A	.	.	.	.	.	.	.	.	.	.	.	.	.	.	.	.	.	.	.	.	.
**Raar_DAB*24**	-	.	.	.	.	.	C	.	A	.	.	.	.	.	.	.	.	.	.	.	.	.	.	.	.	.	.	.	.	.	.	.	.	.	.	.	.	.	.	.	.	.	.	.	.	.	.	.	.	.	.	.	.	.	.	.	.	.	S	.	.	.	.	.	.	.	.	.	.	.	.	.	.	.	.	.	.	.	.	.	.	.	.	.	.	.	.	.	.	.	.
**Raar_DAB*25**	-	.	.	.	.	.	C	.	A	.	.	.	.	.	.	.	.	.	.	.	.	.	.	.	.	.	.	.	.	.	.	.	.	.	.	.	.	.	.	.	.	.	F	.	.	.	.	.	.	.	.	.	.	.	.	Y	.	.	S	.	.	.	.	.	.	.	.	.	.	A	.	.	.	.	.	.	.	.	.	.	.	.	.	.	.	.	.	.	.	.	.
**Raar_DAB*26**	-	.	.	.	.	.	C	.	A	.	.	.	.	.	.	.	.	.	.	.	.	.	.	.	.	.	.	.	.	.	.	.	Y	.	.	.	.	.	.	.	.	.	F	.	.	.	.	.	.	.	.	.	.	.	.	.	.	.	S	.	.	.	.	.	.	.	.	.	.	.	.	.	.	.	.	.	.	.	.	.	.	.	.	.	.	.	.	.	.	.	.
**Raar_DAB*27**	-	.	.	.	.	.	G	.	.	.	.	.	.	.	.	.	.	.	.	.	.	.	.	.	.	.	.	.	.	.	.	.	L	.	.	.	.	.	.	.	.	.	F	.	.	.	.	.	.	.	.	.	.	.	.	Y	.	.	S	.	.	.	.	.	.	R	F	.	.	E	.	.	.	.	.	.	.	.	.	.	.	.	.	.	.	.	.	.	.	.	.
**Raar_DAB*28**	-	.	.	.	.	.	C	.	A	.	.	.	.	.	.	.	.	.	.	.	.	.	.	.	.	.	.	.	.	.	.	.	.	.	.	.	.	.	.	.	.	.	.	.	.	.	.	.	.	.	.	.	S	.	.	.	.	.	.	.	.	.	.	.	.	.	.	.	.	.	.	.	.	.	.	.	.	.	.	.	.	.	.	.	.	.	.	.	.	.	.
**Raar_DAB*29**	-	.	.	.	.	.	G	.	.	.	.	.	.	.	.	.	.	.	.	.	.	.	.	.	.	.	.	.	.	.	.	.	.	.	.	.	.	.	.	.	.	.	.	.	.	.	.	.	.	.	.	.	.	.	.	.	.	.	.	.	.	.	.	.	.	.	.	.	.	.	.	.	.	.	.	.	.	.	.	.	.	.	.	.	.	.	.	.	.	.	.
**Raar_DAB*30**	-	.	.	.	.	.	G	.	A	.	.	.	.	.	.	.	.	.	.	.	.	.	.	.	.	.	.	.	.	.	.	.	.	.	.	.	.	.	.	.	.	.	.	.	.	.	.	.	.	.	.	.	.	.	.	Y	.	.	S	.	.	.	.	.	.	R	F	.	.	E	.	.	.	.	.	.	.	.	.	.	.	.	.	.	.	.	.	.	.	.	.
**Raar_DAB*31**	-	.	.	.	.	.	C	.	A	.	.	.	.	.	.	.	.	.	.	.	.	.	.	.	.	.	.	.	.	.	.	.	.	.	.	.	.	.	.	.	.	.	.	.	.	.	.	.	.	.	.	.	S	.	.	.	.	.	S	.	.	.	.	.	.	.	.	.	.	.	.	.	.	.	.	.	.	.	.	.	.	.	.	.	.	.	.	.	.	.	.
**Raar_DAB*32**	-	.	.	.	.	.	G	.	.	.	.	.	.	.	.	.	.	.	.	.	.	.	L	S	.	.	.	.	.	R	.	.	L	.	.	.	.	.	.	.	.	.	F	.	.	.	.	.	.	.	.	.	.	.	.	Y	.	.	S	.	.	.	.	.	.	R	F	.	.	E	.	.	.	.	.	.	.	.	.	.	.	.	.	.	.	.	.	.	.	.	.
**Raar_DAB*33**	-	.	.	.	.	.	C	.	.	.	.	.	.	.	.	.	.	.	.	.	.	.	.	.	.	.	.	.	.	.	.	.	.	.	.	.	.	.	.	.	.	.	F	.	.	.	.	.	.	.	.	.	.	.	.	Y	.	.	S	.	.	.	.	.	.	.	.	.	.	.	.	.	.	.	.	.	.	.	.	.	.	.	.	.	.	.	.	.	.	.	.
**Raar_DAB*34**	-	.	.	.	.	.	G	.	A	.	.	.	.	.	.	.	.	.	.	.	.	.	.	.	.	.	.	.	.	.	.	.	L	.	.	.	.	.	.	.	.	.	F	.	.	.	.	.	.	.	.	.	.	.	.	Y	.	.	S	.	.	.	.	.	.	R	F	.	.	E	.	.	.	.	.	.	.	.	.	.	.	.	.	.	.	.	.	.	.	.	.
**Raar_DAB*35**	-	.	.	.	.	.	C	.	A	.	.	.	.	.	.	.	.	.	.	.	.	.	.	.	.	.	.	.	.	.	.	.	L	.	.	.	.	.	.	.	.	.	.	.	.	.	.	.	.	.	.	.	.	.	.	Y	.	.	S	.	.	.	.	.	.	R	F	.	.	E	.	.	.	.	.	.	.	.	.	.	.	.	.	.	.	.	.	.	.	.	.
**Raar_DAB*36**	-	.	.	.	.	.	G	.	A	.	.	.	.	.	.	.	.	.	.	.	.	.	.	.	.	.	.	.	.	.	.	.	.	.	.	.	.	.	.	.	.	.	.	.	.	.	.	.	.	.	.	.	S	.	.	.	.	.	.	.	.	.	.	.	.	.	.	.	.	.	.	.	.	.	.	.	.	.	.	.	.	.	.	.	.	.	.	.	.	.	.
**Raar_DAB*37**	-	.	.	.	.	.	G	.	.	.	.	.	.	.	.	.	.	.	.	.	.	.	.	.	.	.	.	.	.	.	.	.	L	.	.	.	.	.	.	.	.	.	.	.	.	.	.	.	.	.	.	.	.	.	.	Y	.	.	S	.	.	.	.	.	.	R	F	.	.	E	.	.	.	.	.	.	.	.	.	.	.	.	.	.	.	.	.	.	.	.	.
**Raar_DAB*38**	-	.	.	.	.	.	C	.	A	.	.	.	.	.	.	.	.	.	.	.	.	.	.	.	.	.	.	.	.	.	.	.	.	.	.	.	.	.	.	.	.	.	.	.	.	.	.	.	.	.	.	.	.	.	.	Y	.	.	S	.	.	.	.	.	.	.	.	.	.	.	.	.	.	.	.	.	.	.	.	.	.	.	.	.	.	.	.	.	.	.	.
**Raar_DAB*39**	-	.	.	.	.	.	G	.	A	.	.	.	.	.	.	.	.	.	.	.	.	.	.	.	.	.	.	.	.	.	.	.	L	.	.	.	.	.	.	.	.	.	.	.	.	.	.	.	.	.	.	.	.	.	.	Y	.	.	S	.	.	.	.	.	.	R	F	.	.	E	.	.	.	.	.	.	.	.	.	.	.	.	.	.	.	.	.	.	.	.	.
**Raar_DAB*40**	-	.	.	.	.	.	G	.	.	.	.	.	.	.	.	.	.	.	.	.	.	.	.	.	.	.	.	.	.	.	.	.	L	.	.	.	.	.	.	.	.	.	F	.	.	.	.	.	.	.	.	.	.	.	.	Y	.	.	S	.	.	.	.	.	.	.	.	.	.	.	.	.	.	.	.	.	.	.	.	.	.	.	.	.	.	.	.	.	.	.	.
**Raar_DAB*41**	-	.	.	.	.	.	G	.	.	.	.	.	.	.	.	.	.	.	.	.	.	.	.	.	.	.	.	.	.	.	.	.	.	.	.	.	.	.	.	.	.	.	F	.	.	.	.	.	.	.	.	.	.	.	.	Y	.	.	S	.	.	.	.	.	.	R	F	.	.	E	.	.	.	.	.	.	.	.	.	.	.	.	.	.	.	.	.	.	.	.	.
**Raar_DAB*42**	-	.	.	.	.	.	C	.	.	.	.	.	.	.	.	.	.	.	.	.	.	.	.	.	.	.	.	.	.	.	.	.	Y	.	.	.	.	.	.	.	.	.	F	.	.	.	.	.	.	.	.	.	.	.	.	.	.	.	S	.	.	.	.	.	.	.	.	.	.	.	.	.	.	.	.	.	.	.	.	.	.	.	.	.	.	.	.	.	.	.	.
**Raar_DAB*43**	-	.	.	.	.	.	C	.	.	.	.	.	.	.	.	.	.	.	.	.	.	.	.	.	.	.	.	.	.	.	.	.	Y	.	.	.	.	.	.	.	.	.	F	.	.	.	.	.	.	.	.	.	.	.	.	Y	.	.	S	.	.	.	.	.	.	.	.	.	.	.	.	.	.	.	.	.	.	.	.	.	.	.	.	.	.	.	.	.	.	.	.
**Raar_DAB*44**	-	.	.	.	.	.	N	.	A	.	.	.	.	.	.	.	.	.	.	.	.	.	.	.	.	.	.	.	.	.	.	.	.	.	.	.	.	.	.	.	.	.	.	.	.	.	.	.	.	.	.	.	.	.	.	.	.	.	.	.	.	.	.	.	.	.	.	.	.	.	.	.	.	.	.	.	.	.	.	.	.	.	.	.	.	.	.	.	.	.	.
**Raar_DAB*45**	-	.	.	.	.	.	N	.	.	.	.	.	.	.	.	.	.	.	.	.	.	.	L	D	.	H	.	.	.	R	.	.	.	.	.	.	.	.	.	.	.	.	F	.	.	.	.	.	.	.	.	.	.	.	.	.	.	.	.	.	.	.	.	.	.	.	.	.	.	.	.	.	.	L	.	.	.	.	.	.	.	.	.	.	.	.	.	.	.	.	.
**Raar_DAB*46**	-	.	.	.	.	.	C	.	A	.	.	.	.	.	.	.	.	.	.	.	.	.	.	.	.	.	.	.	.	.	.	.	.	.	.	.	.	.	.	.	.	.	.	.	.	.	.	.	.	.	.	.	.	.	.	.	.	.	.	.	.	.	.	.	.	.	.	.	.	.	.	.	.	.	.	.	.	.	.	.	.	S	.	.	.	.	.	.	.	.	.
**Raar_DAB*47**	-	.	.	.	.	.	N	.	.	.	.	.	.	.	.	.	.	.	.	.	.	.	L	D	.	H	.	.	.	R	.	.	.	.	.	.	.	.	.	.	.	.	.	.	.	.	.	.	.	.	.	.	.	.	.	Y	.	.	S	.	.	.	.	.	.	.	.	.	.	.	.	.	.	L	.	.	.	.	.	.	.	.	.	.	.	.	.	.	.	.	.
**Raar_DAB*48**	-	.	.	.	.	.	N	.	.	.	.	.	.	.	.	.	.	.	.	.	.	.	.	.	.	.	.	.	.	.	.	.	.	.	.	.	.	.	.	.	.	.	.	.	.	.	.	.	.	.	.	.	.	.	.	.	.	.	S	.	.	.	.	.	.	.	.	.	.	.	.	.	.	.	.	.	.	.	.	.	.	.	.	.	.	.	.	.	.	.	.
**Raar_DAB*49**	-	.	.	.	.	.	N	.	.	.	.	.	.	.	.	.	.	.	.	.	.	.	L	D	.	H	.	.	.	R	.	.	.	.	.	.	.	.	.	.	.	.	F	.	.	.	.	.	.	.	.	.	.	.	.	.	.	.	S	.	.	.	.	.	.	.	.	.	.	.	.	.	.	L	.	.	.	.	.	.	.	.	.	.	.	.	.	.	.	.	.
**Raar_DAB*50**	-	.	.	.	.	.	N	.	.	.	.	.	.	.	.	.	.	.	.	.	.	.	.	.	.	.	.	.	.	.	.	.	.	.	.	.	.	.	.	.	.	.	.	.	.	.	.	.	.	.	.	.	.	.	.	.	.	.	.	.	.	.	.	.	.	.	.	.	.	.	.	.	.	L	.	.	.	.	.	.	.	.	.	.	.	.	.	.	.	.	.
**Raar_DAB*51**	-	.	.	.	.	.	N	.	.	.	.	.	.	.	.	.	.	.	.	.	.	.	L	D	.	H	.	.	.	R	.	.	.	.	.	.	.	.	.	.	.	.	.	.	.	.	.	.	.	.	.	.	.	.	.	.	.	.	.	.	.	.	.	.	.	.	.	.	.	.	.	.	.	.	.	.	.	.	.	.	.	.	.	.	.	.	.	.	.	.	.
**Raar_DAB*52**	-	.	.	.	.	.	G	.	.	.	.	.	.	.	.	.	.	.	.	.	.	.	.	.	.	.	.	.	.	.	.	.	.	.	.	.	.	.	.	.	.	.	.	.	.	.	.	.	.	.	.	.	S	.	.	.	.	.	.	.	.	.	.	.	.	.	.	.	.	.	.	.	.	.	.	.	.	.	.	.	.	.	.	.	.	.	.	.	.	.	.
**Raar_DAB*53**	-	.	.	.	.	.	N	.	A	.	.	.	.	.	.	.	.	.	.	.	.	.	L	A	.	.	.	.	.	.	.	.	.	.	.	.	.	.	.	.	.	.	.	.	.	.	.	.	.	.	.	.	S	.	.	Y	.	.	S	D	.	.	.	.	.	.	.	.	.	.	.	.	.	.	.	.	.	.	.	.	.	.	.	.	.	.	.	.	.	.	.
**Raar_DAB*54**	-	.	.	.	.	.	N	.	.	.	.	.	.	.	.	.	.	.	.	.	.	.	L	D	.	H	.	.	.	.	.	.	.	.	.	.	.	.	.	.	.	.	F	.	.	.	.	.	.	.	.	.	S	.	.	Y	.	.	S	D	.	.	.	.	.	.	.	.	.	E	.	.	.	L	.	.	.	.	.	.	.	.	.	.	.	.	.	.	.	.	.
**Raar_DAB*55**	-	.	.	.	.	.	Y	.	.	.	.	.	.	.	.	.	.	.	.	.	.	.	L	D	.	H	.	.	.	.	.	.	Y	.	.	.	.	.	.	.	.	.	F	.	.	.	.	.	.	.	.	.	S	.	.	Y	.	.	S	D	.	.	.	.	.	R	K	.	.	E	.	.	.	L	.	.	.	.	.	.	.	.	.	.	.	.	.	.	.	.	.
**Raar_DAB*56**	-	.	.	.	.	.	N	.	A	.	.	.	.	.	.	.	.	.	.	.	.	.	L	D	.	H	.	.	.	R	.	.	.	.	.	.	.	.	.	.	.	.	.	.	.	.	.	.	.	.	.	.	.	.	.	.	.	.	.	.	.	.	.	.	.	.	.	.	.	.	.	.	.	.	.	.	.	.	.	.	.	.	.	.	.	.	.	.	.	.	.
**Raar_DAB*57**	-	.	.	.	.	.	G	.	.	.	.	.	.	.	.	.	.	.	.	.	.	.	.	.	.	.	.	.	.	R	.	.	L	.	.	.	.	.	.	.	.	.	F	.	.	.	.	.	.	.	.	.	.	.	.	Y	.	.	S	.	.	.	.	.	.	R	F	.	.	E	.	.	.	.	.	.	.	.	.	.	.	.	.	.	.	.	.	.	.	.	.
