# Supplementary material for: Signatures of historical selection on MHC reveal different selection patterns in the moor frog (Rana arvalis)
Source: Immunogenetics. 2018 Feb 1;70(7):477–84. doi: 10.1007/s00251-017-1051-1 (PMC6006221; doi:10.1007/s00251-017-1051-1)
Supplement: Supplementary file 4 — (PDF 104 kb) [file 251_2017_1051_MOESM4_ESM.pdf]

Table S1. Genetic variation at the MHC II exon 2 locus in the populations presented in (Cortazar-Chinarro et al. 2017). Pond coordinates, n = number of individuals; NA = alleles within a population; As = alleles within a sampling area; PA = private alleles; Ho = observed heterozygosity, He = expected heterozygosity; AR = allelic richness. The HO that deviate significantly from H-W expectations are marked with a *.

Locality	Sampling area	Coordinates		n	NA	A_s	PA	H_O	H_E	AR
**Altwarmbüchen**		52°25'15.81"N	9°51'27.71"E	14	8		3	0.35*	0.85	7.53
**Mardorf**	Germany	52°30'31.2"N	9°19'44.4"E	10	8	17	3	0.30*	0.87	8
**Seebeekwiesen**		52°27'52.9"N	10°05'03.8"E	10	9		2	0.50*	0.91	9
**Sjöhusen**		55°32'42.40"N	13°16'24.53"E	20	14		2	0.8	0.68	4.9
**Tvedöra**	Skåne	55°42'0.81"N	13°25'51.54"E	20	6	18	7	0.9	0.86	10.09
**Räften**		55°43'17.34"N	13°17'3.02"E	19	6		1	0.42*	0.54	4.62
**Österbybruk**		60°10'42.09"N	17°51'16.06"E	18	11		5	0.83	0.87	8.49
**Valsbrunna**	Uppsala	59°45'14.66"N	17° 2'8.95"E	19	11	19	7	0.78	0.79	8.07
**Holmsjön**		63°45'27.21"N	20°24'22.02"E	19	5		0	0.57	0.64	4.01
**Nydalasjön**	Umeå	63°49'23.44"N	20°20'1.60"E	19	9	10	3	0.57*	0.76	6.63
**Besbyn**		65°41'5.19"N	22°12'43.80"E	20	4		2	0.3	0.31	2.98
**Ernäs**	Luleå	65°31'14.69"N	21°41'12.78"E	19	4	6	2	0.42	0.44	3.05
				207			37	0.56	0.71	6.45
