# Supplementary material for: Signatures of historical selection on MHC reveal different selection patterns in the moor frog (Rana arvalis)
Source: Immunogenetics. 2018 Feb 1;70(7):477–84. doi: 10.1007/s00251-017-1051-1 (PMC6006221; doi:10.1007/s00251-017-1051-1)
Supplement: Supplementary file 5 — (PDF 29.1 kb) [file 251_2017_1051_MOESM5_ESM.pdf]

Table S2. Comparation of different methods to identity codons for which signal of positive selection (dN/dS ratio) is strongest. Significant positively selective codons have been calculated using OmegaMap (Wilson and McVean 2006), Selecton server (Stern et. al 2007) and CodeML (PAML, version 4.9d) (Zang 2017) and are marked with an X, the numbers represent the amino acid position in the MHC class II molecule.

	**Positively selected codons in Omegamap**													
	**11**	**13**	**27**	**28**	**34**	**37**	**47**	**57**	**60**	**63**	**70**	**71**	**74**	**78**
**Nothern cluster**														
		X				X	X		X	X			X	
**Southern cluster**														
	X	X	X	X	X		X		X	X	X		X	X
	**Positively selected codons Selecton server**													
**Nothern cluster**														
		X		X		X				X	X	X		
**southern cluster**														
	X	X			X	X	X		X	X	X	X		X
	**Positively selected codons CodeML (PALM)**													
**Nothern cluster**														
Model 2		X	X	X		X	X	X	X	X	X	X	X	
Model 8		X	X	X		X	X			X	X	X		
**southern cluster**														
Model 2	X	X	X	X	X	X	X	X	X	X	X	X	X	X
Model 8	X	X		X	X	X	X	X	X	X	X	X	X	X
Distance to Bondinas et. Al 2007	**0**	**0**	**1**	**0**	**4**	**0**	**0**	**0**	**1**	**2**	**0**	**1**	**0**	**0**
