# Supplementary material for: Signatures of historical selection on MHC reveal different selection patterns in the moor frog (Rana arvalis)
Source: Immunogenetics. 2018 Feb 1;70(7):477–84. doi: 10.1007/s00251-017-1051-1 (PMC6006221; doi:10.1007/s00251-017-1051-1)
Supplement: Supplementary file 6 — (PDF 89.1 kb) [file 251_2017_1051_MOESM6_ESM.pdf]

Table S3. Number of recombination events calculated by different methods, implemented in the RDP package (Martin et al. 2010): RDP (Martin and Rybicki 2000), GENECONV (Padidam et al. 1999), Maxchi (Maynard Smith 1992), Bootscan (Salminen et al 1995), Chimera (Posada and Crandall 2001), and number of recombination breakpoints calculated in GARD (Kosakovsky Pond et al. 2006) implemented in Datamonkey (Delport et al. 2010)

	**No. of sequences**	**RDP**	**GENECONV**	**Maxchi**	**Bootscan**	**Chimera**	**GARD**
**Entire gradient**	57	0	0	1	0	1	0
**Northern cluster**	14	0	0	0	0	0	0
**Southern Cluster**	47	0	0	1	0	1	0

References:

Delport W, Poon AFY, Frost SDW, Pond SLK (2010) Datamonkey 2010: a suite of phylogenetic analysis tools for evolutionary biology. Bioinformatics 26: 2455-2457.

Kosakovsky Pond SL, Posada D, Gravenor MB, Woelk CH, Frost SDW (2006) Automated phylogenetic detection of recombination using a genetic algorithm. Mol Biol Evol 23: 1891-1901.

Martin D, Rybicki E (2000) RDP: detection of recombination amongst aligned sequences. *Bioinformatics* 16: 562-563.

Martin DP, Lemey P, Lott M, *et al.* (2010) RDP3: a flexible and fast computer program for analyzing recombination. *Bioinformatics* 26: 2462-2463.

Maynard Smith J (1992) Analyzing the mosaic structure of genes. J Mol Evol 34**:** 126-129.

Padidam M, Sawyer S, Fauquet CM (1999) Possible emergence of new geminiviruses by frequent recombination. *Virology* 265: 218-225.

Posada D, Crandall KA (2001) Evaluation of methods for detecting recombination from DNA sequences: Computer simulations. Proc Natl Acad Sci U S A 98: 13757-13762.

Salminen MO, Carr JK, Burke DS, McCutchan FE. 1995. Identification of breakpoints in intergenotypic recombinants of HIV type 1 by BOOTSCANning. *AIDS Res Hum Retroviruses* 11:1423-1425.
